# Ophthalmic Immune-Related Adverse Events in Cancer Immunotherapy: Tissue-Specific Mechanisms, Clinical Phenotypes, and Consensus-Based Management

**DOI:** 10.3390/ijms27135944

**Published:** 2026-07-01

**Authors:** Yuan Zong, Mingming Yang, Jing Zhang, Yaru Zou, Zizhen Ye, Jiaxin Deng, Wendong Gu, Kyoko Ohno-Matsui, Koju Kamoi

**Affiliations:** Department of Ophthalmology & Visual Science, Graduate School of Medical and Dental Sciences, Institute of Science Tokyo, Tokyo 113-8510, Japan; zongyuan666@gmail.com (Y.Z.); yangmm-12@outlook.com (M.Y.); zhangj.c@foxmail.com (J.Z.); alicezouyaru519@gmail.com (Y.Z.); yezizhen518@gmail.com (Z.Y.); dengjiaxin.med@gmail.com (J.D.); gwd981107@gmail.com (W.G.); k.ohno.oph@tmd.ac.jp (K.O.-M.)

**Keywords:** immune checkpoint inhibitors, ophthalmic immune-related adverse events, uveitis, ocular surface disease, optic neuritis, biomarkers

## Abstract

Immune checkpoint inhibitors (ICIs) have transformed cancer therapy by restoring anti-tumor immunity, but immune activation can disrupt ocular immune homeostasis and induce ophthalmic immune-related adverse events (OirAEs). Although uncommon, OirAEs may involve nearly all ocular compartments and can cause irreversible visual impairment or interruption of effective anticancer therapy. The 2025 international consensus criteria now provide a standardized framework for defining and classifying OirAEs. This review integrates current evidence on ICI-associated ocular toxicity, with emphasis on tissue-specific immune mechanisms and their clinical implications. Blockade of the PD-1/PD-L1 and CTLA-4 pathways may impair ocular immune privilege, expand autoreactive T-cell subsets, alter cytokine and chemokine networks, and amplify autoantibody-mediated retinal injury. These processes provide a plausible framework for understanding diverse phenotypes, including uveitis, ocular surface disease, optic neuritis, orbital inflammation, ocular myopathy, and retinopathy. We also outline a mechanism-informed management approach that balances visual preservation with maintenance of systemic anti-tumor immunity. Local corticosteroid therapy, cautious systemic immunosuppression, and selected steroid-sparing biologics should be individualized according to severity, anatomical involvement, and the oncologic context. Together, these insights support a consensus-based and mechanism-informed framework for recognizing and managing OirAEs while preserving systemic anti-tumor immunity.

## 1. Introduction

Since their first clinical approval in 2011, immune checkpoint inhibitors (ICIs) have transformed the treatment landscape for a broad range of malignancies, with durable tumor responses and long-term survival benefits observed in subsets of patients across cancer types. Rather than directly targeting tumor cells, ICIs function by blocking inhibitory immune checkpoint signaling pathways to reinvigorate T-cell-mediated anti-tumor immunity; this same attenuation of physiological immune tolerance mechanisms can also predispose patients to a wide spectrum of inflammatory immune-related adverse events (irAEs) [1]. As ICI indications continue to expand, irAEs have emerged as a core clinical challenge, with severe events requiring discontinuation of potentially life-saving anti-tumor therapy in a subset of treated patients.

Ocular irAEs (OirAEs) are a relatively rare but clinically important subset of these events, occurring in approximately 1% of ICI recipients, and are frequently overlooked in routine oncology follow-up due to their non-specific early symptoms [2]. While most events are mild, they can cause significant morbidity including permanent vision loss, with uveitis representing the most common sight-threatening subtype (incidence 0.3–3% across ICI cohorts) [3,4]. Beyond uveitis, ICIs can induce a range of other ocular toxicities, including dry eye disease, conjunctivitis, keratitis, retinopathies, neuro-ophthalmic events [5,6,7]. These toxicities are thought to arise, at least in part, from disruption of ocular immune privilege and activation of autoreactive immune responses against tissue-specific ocular antigens, with potentially distinct pathogenic mechanisms contributing to damage in different anatomical compartments.

Prior to 2025, progress in OirAE research and clinical management was significantly hampered by the absence of standardized disease definitions and classification frameworks, which limited cross-study comparability and development of evidence-based treatment guidelines. This gap was recently addressed by an international expert panel, which published the first consensus diagnostic and management criteria for 19 distinct OirAE entities via a modified Delphi process. In this review, we integrate this 2025 consensus classification to provide a comprehensive overview of OirAE pathogenic mechanisms, clinical phenotype spectrum, and multidisciplinary management strategies, with particular emphasis on molecular drivers of tissue-specific toxicity as a model for understanding organ-specific irAE pathogenesis more broadly. Our overarching goal is to improve clinical recognition of these events and inform precision therapeutic strategies that preserve visual function without compromising anti-tumor efficacy.

## 2. Overview of Immune Checkpoint Inhibitors

**Figure 1:**
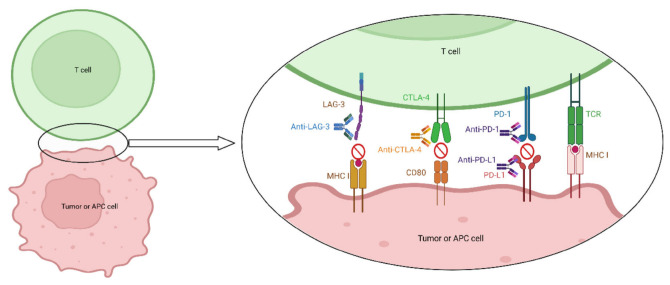
Mechanism of action of immune checkpoint inhibitors.

ICIs are monoclonal antibodies that block inhibitory immune checkpoint receptors or their ligands, thereby enhancing T-cell-mediated anti-tumor immunity. Several classes of ICIs have been approved internationally, including antibodies targeting CTLA-4, PD-1, PD-L1, and LAG-3 (Table 1). Anti-CTLA-4 antibodies (ipilimumab, tremelimumab) block the interaction between CTLA-4 on T cells and B7 (CD80/CD86) on antigen-presenting cells, preventing CTLA-4 from outcompeting CD28 for B7 binding. Beyond checkpoint blockade, anti-CTLA-4 antibodies also deplete intratumoral regulatory T cells through Fc-dependent mechanisms—a property that contributes to their distinct toxicity profile [8,9]. Anti-PD-1 antibodies (nivolumab, pembrolizumab, cemiplimab, toripalimab, dostarlimab) and anti-PD-L1 antibodies (atezolizumab, durvalumab, avelumab, envafolimab, sugemalimab) disrupt the PD-1/PD-L1 axis that normally limits T-cell effector function in peripheral tissues. Unlike CTLA-4, which acts during early T-cell priming, PD-1 regulates later-stage responses, and PD-L1 is broadly expressed across normal tissues—including the eye—making this pathway particularly relevant to ocular irAEs [10,11]. Anti-LAG-3 antibodies (relatlimab) target a third inhibitory receptor on effector T cells and Tregs; relatlimab was approved in 2022 in combination with nivolumab for melanoma, marking LAG-3 as a clinically validated fourth checkpoint target [12]. Since the first ICI approval in 2011, these agents have transformed cancer outcomes, but their shared mechanism—removing inhibitory signals that restrain T-cell activation—can predispose patients to immune-related adverse events across nearly every organ system, including the eye [1]. The molecular mechanisms underlying ICI-mediated T-cell activation are summarized in Figure 1, which illustrates the three core checkpoint pathways targeted by currently approved agents: CTLA-4, PD-1/PD-L1, and LAG-3. Blockade of these pathways disinhibits anti-tumor T-cell responses, but also disrupts immune tolerance in peripheral tissues including the eye, providing the mechanistic foundation for the ophthalmic immune-related adverse events discussed in subsequent sections.

Mechanism of action of immune checkpoint inhibitors. The image illustrates how immune checkpoint inhibitors (ICIs) block inhibitory pathways to enhance T-cell activation against tumor cells. Left: Overview of T-cell interaction with a tumor or antigen-presenting cell (APC). Right: Detailed molecular interactions showing three major checkpoint pathways targeted by ICIs: CTLA-4/CD80, PD-1/PD-L1, and LAG-3/MHC II. Anti-CTLA-4 antibodies prevent CTLA-4 from competing with CD28 for binding to B7 molecules (CD80), promoting T-cell activation. Anti-PD-1 and anti-PD-L1 antibodies block the PD-1/PD-L1 interaction that normally inhibits T-cell function in peripheral tissues. Anti-LAG-3 antibodies prevent LAG-3 binding to MHC II, removing another inhibitory signal. By blocking these immune checkpoints, ICIs restore T-cell function and enhance anti-tumor immune responses, which can also lead to immune-related adverse events in various tissues, including the eye. (Created in https://BioRender.com).

## 3. Molecular Mechanisms Underlying the Tissue Specificity of Ocular Immune-Related Adverse Events

Ocular irAEs span from common dry eye and anterior uveitis to rarer but sight-threatening optic neuritis and panuveitis. This heterogeneity—and the fact that different ICIs produce different ocular phenotypes—points to a layered immunological cascade: differential PD-L1 expression across ocular tissues, disruption of immune privilege, selective activation of distinct T-cell and B-cell subsets, and compartment-specific cytokine release [26,27]. Decoding this cascade is the prerequisite for predictive biomarkers and mechanism-based therapy.

### 3.1. PD-L1 Expression Across Ocular Tissues as a Determinant of Immune Privilege

The eye maintains immune privilege through anatomical barriers, immunosuppressive soluble factors, and constitutive expression of surface molecules that actively suppress local immune responses [28]. Among these, PD-L1 (B7-H1) is the dominant checkpoint ligand. Flow cytometry and immunohistochemistry of normal human eyes have shown PD-L1 on corneal epithelial and endothelial cells, iris/ciliary body cells, and retinal pigment epithelial (RPE) cells [29]. About 92% of RPE cells constitutively express PD-L1, and IFN-γ stimulation upregulates it further on all PD-L1-positive ocular cell types [29]. Hori et al. further confirmed that PD-L1 is constitutively expressed on endothelial cells of the cornea, stromal cells, the iris-ciliary body, and the neural retina, and that PD-L1 expressed in the cornea induces apoptosis of PD-1-expressing T cells through a contact-dependent mechanism. The functional significance of this pathway is underscored by the observation that blockade of PD-L1 or PD-1 with neutralizing antibodies intensifies the rejection reaction after corneal transplantation [28].

PD-L2, by contrast, is undetectable in normal or inflamed ocular tissues [29]. This makes PD-L1 the dominant immune checkpoint ligand in the eye. This proposed PD-L1 gradient may help contextualize the observed OirAE spectrum, although direct clinical validation in ICI-treated ocular tissues remains limited. This PD-L1 gradient model is consistent with the broader ocular immune checkpoint landscape, which additionally involves TIM-3, LAG-3, and CEACAM1 in regulating ocular immune responses [30].

### 3.2. Disruption of Ocular Immune Privilege by Immune Checkpoint Inhibitors

ICIs can disrupt this PD-1/PD-L1 axis through two routes. Anti-PD-1/PD-L1 antibodies directly block the receptor–ligand interaction, removing the brake that keeps T cells quiescent within ocular tissues. Anti-CTLA-4 antibodies have a more pronounced effect on the regulatory compartment: CTLA-4 (CD152) is constitutively expressed on Tregs and normally suppresses CD80/CD86 co-stimulation on antigen-presenting cells [31]. Blocking CTLA-4 causes Fc-dependent Treg depletion in the tumor microenvironment and sharply reduces the Treg-to-effector T-cell ratio—an effect that may also be relevant to inflamed ocular tissues, although direct ocular evidence remains limited [31].

The consequence is loss of the immunosuppressive intraocular milieu. In the normal cornea, PD-L1/PD-1-mediated T-cell apoptosis occurs across epithelium, stroma, and endothelium, deleting effector T cells and preventing immune rejection [28,32]. RPE cells express high PD-L1 basally, contributing to immune privilege. ICI-related uveitis likely reflects reduced self-tolerance after PD-1/PD-L1 blockade [26]. Between direct checkpoint blockade and Treg dysfunction, a permissive environment emerges for autoreactive T-cell clones to infiltrate ocular tissues.

### 3.3. T-Cell Subset Activation and Clonal Expansion in Ocular irAEs

When ocular immune privilege is disrupted, the subsequent inflammation is driven by distinct T-cell subsets that target different ocular compartments through different molecular pathways.

Single-cell RNA sequencing and TCR profiling of 186,435 immune cells from 17 ICI-treated patients identified a clonally expanded subset of CD4+ cytotoxic T lymphocytes (CD4+ CTLs) with a distinct effector phenotype that was specifically enriched in patients with immune-related adverse events (enrichment score 2.52 versus 0 in controls, *p* < 0.05). These cells highly expressed CXCR3 (FC = 2.03, FDR = 7.7 × 10^−4^), the chemokine receptor for CXCL10 (IP-10), which has been previously nominated as a serum biomarker for severe ICI-induced toxicity [33]. Although the study focused on neurological adverse events, the mechanistic finding may be relevant to ocular inflammation, particularly given the detection of CXCL10 in ocular fluids from selected ICI-induced inflammatory cases.

The “shared antigen” hypothesis has been proposed to explain why uveal tissues are preferentially targeted. Uveal melanocytes share developmental origin and antigenic repertoire with cutaneous melanocytes. Although direct evidence from ocular tissues in ICI-treated patients remains limited, it is hypothesized that when ICIs reinvigorate T cells against melanoma, expanded CD8+ clones directed against shared melanocytic antigens—Melan-A (MART-1), tyrosinase-related proteins—may infiltrate the choroid and retina [32,34]. Weak PD-L1 expression in the iris and ciliary body [29] combined with abundant autoantigens in melanocyte-rich uveal tissue may render the uveal tract especially susceptible. The clinical presentation resembles Vogt–Koyanagi–Harada (VKH) disease, a classic T-cell-mediated autoimmune disorder.

These observations suggest that ICI-induced uveitis may represent a consequence of immune disinhibition against shared tumor–ocular antigens, rather than a classical drug hypersensitivity. Consistent with this hypothesis, anterior uveitis rates are highest with ipilimumab-containing regimens that combine checkpoint blockade with Treg depletion [35].

The Th17-IL-17A axis has been implicated in ICI-associated dry eye disease. Th17 cells secrete IL-17A, which recruits neutrophils and promotes granulomatous lacrimal infiltration, and IL-22, which disrupts ocular surface epithelial barrier function [26]. This non-cytotoxic mechanism differs fundamentally from the cytotoxic damage in uveitis and may help explain why dry eye disease is frequently observed among OirAEs.

The Th1-IFN-γ-CXCL10 axis has been proposed as a driver of optic neuritis and retinal vasculitis based on elevated CXCL10 levels in the aqueous humor of ICI-treated patients [33,36] and mechanistic parallels with autoimmune neuritis. Activated Th1 cells may infiltrate the optic nerve and retinal vessels, releasing IFN-γ that could trigger demyelination, axonal injury, and vascular inflammation. The optic nerve is normally protected by the blood–brain barrier, but its very low PD-L1 expression [28] may offer little resistance once immune cells cross this barrier.

### 3.4. Cytokine and Chemokine Effector Profiles in Ocular Fluids

Anti-PD-1-related uveitis shares cytokine profiles with non-infectious uveitis: elevated IL-6, IL-17, TNF-α, and IFN-γ. Among metastatic melanoma patients on ICI therapy, severe irAEs coincide with elevated levels of eleven cytokines—fractalkine, FGF-2, IFN-α2, IL-12p70, IL-1α, IL-1β, IL-1RA, IL-2, IL-13, G-CSF, and GM-CSF. Serum IL-2, IP-10 (CXCL10), and MCP-4 (CCL13) correlate with favorable anti-PD-1 outcomes, and elevated IP-10/CXCL10 signals irAE risk in renal cell carcinoma [26].

Limited ocular fluid data provide direct supportive evidence for this pathway. Yoshida et al. [36] analyzed aqueous and vitreous humor from a patient with nivolumab-induced panuveitis and found IL-6, G-CSF, and CXCL10 (IP-10) markedly elevated compared to controls. This simultaneous elevation of G-CSF, an innate immune amplifier, and CXCL10, a Th1-associated chemokine, points to synergistic innate–adaptive crosstalk in ICI-related uveitis. Furthermore, multiplex analysis of aqueous humor from eyes with non-infectious uveitis has consistently shown elevated levels of IL-6, IL-8, MCP-1, and MIP-1β [37], confirming that these mediators are central to the intraocular inflammatory response.

These cytokine signatures may have therapeutic implications. IL-6 receptor blockade, such as tocilizumab, has been reported as a potential option in selected non-infectious uveitis and ICI-associated inflammatory cases. Anti-TNF-α agents, including infliximab and adalimumab, may also be considered in selected refractory inflammatory phenotypes, particularly when TNF-α-mediated inflammation is suspected. However, because OirAE-specific evidence remains limited, these approaches should be individualized through multidisciplinary assessment with careful consideration of systemic anti-tumor immunity [26].

### 3.5. Autoantibody-Mediated Mechanisms and Paraneoplastic Associations

T-cell-mediated mechanisms appear to play a major role in uveitic inflammation, whereas humoral immunity— often overlooked in OirAE discussions—plays its own distinct role. ICI therapy can boost pre-existing autoantibody production or trigger epitope spreading, producing ocular complications mechanistically separate from T-cell-mediated uveitis [32].

A representative example is melanoma-associated retinopathy (MAR). Unlike the direct cellular cytotoxicity of uveitis, MAR is antibody-driven: antiretinal antibodies (e.g., against bipolar cells) cross-react between tumor and retinal antigens [38,39]. ICIs may amplify pre-existing or newly emerging pathogenic antibodies, potentially contributing to retinal dysfunction even when systemic tumor control is achieved [40,41]. Cancer-associated retinopathy (CAR), mediated by antibodies against recoverin and other photoreceptor proteins, may involve a similar autoantibody-mediated mechanism [39,40]. This mechanistic distinction, while simplified, may help guide diagnostic and therapeutic decisions: T-cell-mediated pathways appear to dominate uveal inflammation, whereas autoantibody-driven processes are more relevant to retinopathy. Recognizing this difference has practical implications for diagnosis and treatment selection.

### 3.6. Synthesis and Clinical Implications

The tissue specificity of ICI-induced ocular irAEs can be conceptualized as a four-step cascade: (1) constitutive PD-L1 expression across ocular tissues establishes a baseline immune-privilege gradient; (2) ICIs disrupt this gradient through direct PD-1/PD-L1 blockade and, for anti-CTLA-4 agents, Fc-dependent Treg depletion; (3) loss of immune privilege permits clonal expansion of T-cell subsets—CD8+ CTLs targeting melanocyte-rich uvea, Th17 cells driving ocular surface inflammation, and Th1 cells mediating neuro-ophthalmic complications—alongside autoantibody production against retinal antigens; and (4) tissue-specific cytokine and chemokine release (IL-6, IL-17, TNF-α, IFN-γ, CXCL10, G-CSF) amplifies and perpetuates the inflammation.

Two additional layers modulate this cascade. First, IgG subclass differences (IgG1 vs. IgG2) alter Fc receptor binding and effector functions, contributing to the distinct irAE profiles of different ICI antibodies [42,43]. Second, ICIs remodel the tumor microenvironment, releasing previously sequestered antigens that can fuel broader systemic immune activation [44,45].

**Figure 2:**
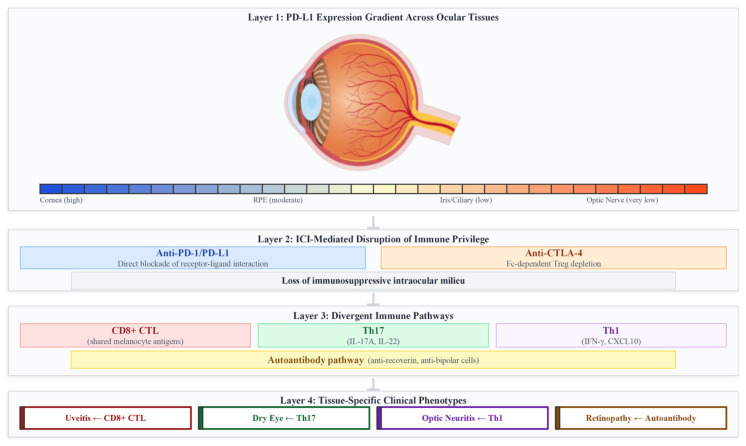
Mechanistic cascade of ocular immune-related adverse events (OirAEs). The cascade is organized into four interconnected layers. Layer 1: Constitutive PD-L1 expression varies across ocular tissues, with a gradient from high expression in the corneal endothelium through moderate expression in the retinal pigment epithelium (RPE) and low expression in the iris and ciliary body to very low expression in the optic nerve. This gradient establishes a baseline immune-privilege hierarchy. Layer 2: Immune checkpoint inhibitors (ICIs) disrupt this hierarchy through two distinct mechanisms: anti-PD-1/PD-L1 antibodies directly block the receptor–ligand interaction, while anti-CTLA-4 antibodies deplete regulatory T cells (Tregs) via Fc-dependent mechanisms. Both pathways converge on loss of the immunosuppressive intraocular milieu. Layer 3: Loss of immune privilege permits the activation of divergent immune pathways. CD8+ cytotoxic T lymphocytes (CTLs) targeting shared melanocyte antigens may contribute to uveitic inflammation; Th17 cells releasing IL-17A and IL-22 have been implicated in ocular surface disease; Th1 cells secreting IFN-γ and CXCL10 may participate in neuro-ophthalmic complications; and autoantibody production (against recoverin, bipolar cell antigens, and other retinal proteins) may contribute to retinopathy. Layer 4: These distinct immune pathways converge on tissue-specific clinical phenotypes: uveitis (potentially CD8+ CTL-associated), dry eye disease (Th17-associated), optic neuritis (Th1-associated), and retinopathy (autoantibody-associated). This mechanistic model was constructed using the Generic Diagramming Platform analytical framework [46]. Abbreviations: PD-L1, programmed death-ligand 1; RPE, retinal pigment epithelium; ICI, immune checkpoint inhibitor; Treg, regulatory T cell; CTL, cytotoxic T lymphocyte; IFN-γ, interferon-gamma; CXCL10, C-X-C motif chemokine ligand 10.

This mechanistic cascade is summarized in Figure 2. Clinically, the IRIS Registry data indicate patients with prior uveitis show recurrence rates of 38.9% (anterior) to 51.1% (intermediate/posterior/panuveitis) after ICI initiation [35]. Pre-treatment ophthalmic evaluation should be considered in these at-risk populations. The distinct molecular signatures of each T-cell subset and antibody pathway offer rational targeting: IL-6 receptor blockade for steroid-refractory uveitis, TNF-α antagonists for scleritis, and potentially IL-17A inhibition for severe dry eye. The next step is to validate tear fluid and aqueous humor cytokine profiles as non-invasive biomarkers and to develop strategies that suppress ocular inflammation—T-cell-driven or antibody-mediated—without blunting the systemic anti-tumor response.

## 4. Incidence and Risk Factors of Ocular Immune-Related Adverse Events: Epidemiology and Classification

Ocular irAEs (OirAEs) associated with ICIs, though rarely life-threatening, can significantly impair visual function and reduce quality of life. A systematic review and meta-analysis of nine clinical trials [47] reported an odds ratio (OR) of 3.40 (95% CI: 1.32–8.71) for all-grade ocular toxicities in ICI recipients across multiple cancer types. Nevertheless, the true incidence of OirAEs is likely underreported because most estimates are derived from retrospective case series and pharmacovigilance databases, where ocular toxicities are inconsistently coded and may escape detection in routine oncology follow-up [48]. Prospective cohort studies employing standardized ophthalmic monitoring are needed to establish more accurate incidence figures.

Risk factors for developing OirAEs include prior ocular inflammatory conditions, ocular trauma or surgery, underlying autoimmune disorders, and renal insufficiency [49,50]. The type of cancer also influences risk, with melanoma associated with a 2–3 fold increased risk (data primarily from retrospective analyses and pharmacovigilance studies with variable denominators) compared to other cancers [51,52]. Lung cancer patients appear more susceptible to myasthenia gravis with ocular involvement [51].

**Figure 3:**
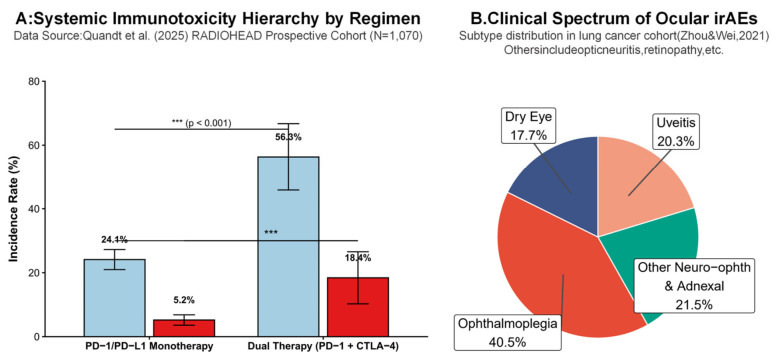
Immunotoxicity hierarchy and ocular clinical spectrum. (**A**) Comparative incidence of systemic immune-related adverse events (irAEs) stratified by ICI regimen. In Panel (**A**), blue bars indicate any-grade systemic irAEs, whereas red bars indicate severe Grade III–IV systemic irAEs. Data derived from the prospective RADIOHEAD cohort (Quandt et al., 2025 [56]) involving 1070 patients. The analysis supports the presence of an immunotoxicity hierarchy: Dual therapy (PD-1 + CTLA-4) induces a >3-fold higher rate of severe toxicity (Grade III-IV, 18.4%) compared to PD-1/PD-L1 monotherapy (5.2%). Error bars represent 95% confidence intervals. *** *p* < 0.001, as reported in the original study. (**B**) The clinical spectrum of ocular irAEs in a representative cohort of lung cancer patients (Adapted from Zhou & Wei, 2021 [51]). The distribution highlights the heterogeneity of ocular manifestations, with ophthalmoplegia (40.5%), uveitis (20.3%), and dry eye syndrome (17.7%) being the most prevalent subtypes. Important caveat: Panels (**A**,**B**) are derived from fundamentally different populations and evidence levels. Panel (**A**) presents systemic irAE rates from the prospective RADIOHEAD cohort study (n = 1070, mixed cancer types, Quandt et al. 2025 [56]) and illustrates the immunotoxicity hierarchy across ICI regimens; these data reflect systemic, not ocular-specific, toxicity. Panel (**B**) presents ocular subtype distribution from a single-center retrospective lung cancer cohort (n = 79, Zhou and Wei 2021 [51]) and may not be generalisable to other cancer types or ICI regimens. The juxtaposition is intended to contrast the systemic risk gradient with the diversity of ocular presentations, not to imply direct comparability between the two data sources. Abbreviations: PD-1, programmed cell death protein 1; PD-L1, programmed death-ligand 1; CTLA-4, cytotoxic T-lymphocyte-associated protein 4.

Regarding ICI class, combination therapy (particularly ipilimumab plus nivolumab) carries the highest risk, while CTLA-4 inhibitors and PD-1/PD-L1 inhibitors may show different predilections for specific ocular structures [53,54,55]. To illustrate this hierarchical risk profile with contemporary real-world evidence, we incorporated data from the prospective RADIOHEAD cohort study by Quandt et al. [56] (Figure 3A). Unlike earlier analyses, this study highlights that dual therapy (PD-1 + CTLA-4) is associated with a more than three-fold increase in severe (Grade III–IV) systemic toxicity (18.4%) compared to PD-1/PD-L1 monotherapy (5.2%). We present these systemic findings to underscore a crucial principle: regimens with stronger systemic immunogenicity warrant heightened ophthalmological vigilance. Regarding the clinical heterogeneity of these events, Figure 3B summarizes the distribution of ocular subtypes based on a representative lung cancer cohort study by Zhou and Wei [51]. Their data indicate that ophthalmoplegia (40.5%) and uveitis (20.3%) are the predominant manifestations. This diverse spectrum highlights the need for clinicians to look beyond common dry eye symptoms and conduct thorough neuro-ophthalmic evaluations. We acknowledge that these two data sources differ in population, study design, and outcome ascertainment. Panel A illustrates the systemic immunotoxicity gradient that informs ophthalmological vigilance recommendations, while Panel B, despite its single-center retrospective design, exemplifies the heterogeneity of ocular presentations that clinicians should anticipate. We caution against direct quantitative comparison between the two panels, as noted in the figure legend.

### ICI-Related Uveitis: Epidemiology, Risk Factors and Clinical Manifestations

Uveitis is among the most common and prognostically significant OirAEs, with potential to cause sight-threatening complications including macular edema, posterior synechiae and elevated intraocular pressure [57]. Delayed diagnosis correlates with increased risk of permanent vision loss, and timely recognition not only preserves visual function but also minimizes unnecessary interruption of life-saving ICI therapy [55,58]. Overall incidence of ICI-related uveitis ranges from 0.3% to 3% across published cohorts (predominantly derived from retrospective chart reviews and pharmacovigilance databases), which is substantially higher than for most other drug-related ocular toxicities. These estimates should be interpreted with caution given variability in cohort composition, ICI regimen, cancer type, ophthalmological surveillance intensity, and diagnostic criteria across studies. Demographic factors including age, sex and ethnicity do not appear to modify risk. Established risk factors include: prior history of ocular inflammation (38.9% recurrence rate for anterior uveitis, 51.1% for intermediate/posterior/panuveitis, vs. 3.6% baseline incidence in patients without prior disease) [35,59], prior ocular surgery/trauma [53], underlying autoimmune disease and renal insufficiency. Prior corticosteroid exposure may confer a protective effect in melanoma cohorts [60]. Cancer type is an independent risk modifier: melanoma is associated with a 2–3-fold higher uveitis risk compared to other malignancies, followed by lung cancer [51,52]. Combination ICI therapy (anti-CTLA-4 + anti-PD-1) carries the highest risk, with anti-CTLA-4 agents specifically associated with more severe phenotypes. Clinical presentation varies by anatomical subtype, classified per the International Uveitis Study Group framework and 2025 consensus diagnostic criteria into anterior, intermediate, posterior and panuveitis, with diagnostic certainty graded as definite/probable/possible based on clinical features, ancillary testing (OCT, fluorescein angiography) and exclusion of alternative etiologies [61]. Anterior uveitis is the most frequent subtype, typically associated with anti-PD-1 monotherapy, presenting with conjunctival injection, pain, photophobia and anterior chamber cells/flare on slit-lamp examination [62,63]. Intermediate uveitis manifests primarily as floaters and vitreous haze, while posterior uveitis and panuveitis are associated with worse visual outcomes, with symptoms including photopsia, scotomata and permanent vision loss in severe cases [64,65,66]. Median presenting visual acuity is ~20/40, with better outcomes in anterior uveitis (20/30) and worse in panuveitis (20/40–20/50); most cases are CTCAE Grade 2 or 3, with Grade 4 events occurring in <16.5% of cases [62,66]. Bilateral involvement has been reported frequently in some series, although the reported frequency varies across cohorts [67]. Distinct phenotypes are linked to specific ICI classes: anti-CTLA-4 therapy has been reported in association with severe VKH-like phenotypes, presenting with bilateral granulomatous panuveitis, serous retinal detachments, choroidal thickening and extraocular features including vitiligo and tinnitus [57]. Rare presentations including birdshot-like chorioretinopathy have also been reported, with strong association with *HLA-A29* in older Caucasian males [68]. Symptom onset occurs at a median of 9 weeks after ICI initiation, with combination therapy associated with earlier onset [62]. The heterogeneous clinical phenotypes of ICI-associated uveitis are underpinned by tissue-specific immunological cascades and immune checkpoint disruption patterns, which are detailed in Section 3.

## 5. Other Ocular Immune-Related Adverse Events

Although uveitis represents one of the most frequently reported and potentially sight-threatening forms of OirAEs, a spectrum of non-uveitic ocular pathologies may also significantly impact patients’ visual function and quality of life, necessitating heightened clinical vigilance. Table 2 summarizes the clinical characteristics of various OirAEs, serving as a practical reference for clinicians.

### 5.1. Neuro-Ophthalmic Events

Neuroimmune-related irAEs may affect a broad spectrum of neuromuscular structures, resulting in neuro-ophthalmic manifestations such as optic neuritis and ocular myopathies that involve ocular motility, pupillary function, visual pathways, or eyelid function. The underlying mechanism is postulated to involve ICIs disrupting immune tolerance within the CNS: ICIs remove inhibitory signals of T-cell activation, enabling autoreactive T cells (e.g., autoreactive CD4+ T cells, cytotoxic CD8+ T cells) to be activated and expanded, which may target optic nerve myelin sheaths or axons [55,71].

ICI-associated optic neuritis is a rare complication characterized by inflammation of the optic nerve, manifesting clinically as bilateral, painless visual decline and visual field defects. Examination typically reveals a relative afferent pupillary defect (RAPD), and fundoscopic evaluation may demonstrate optic disk edema (papillitis) or normal findings (retrobulbar optic neuritis) [6].

Ocular myopathies frequently present with uni- or bilateral ophthalmoplegia, with patients commonly reporting diplopia, ptosis, or abnormal ocular positioning. This section covers non-uveitic OirAEs per the 2025 consensus classification, which divides ocular irAEs into two major categories: neuro-ophthalmic/orbital disorders and ocular surface/retinal disorders. Chang et al. [61] consensus framework further distinguishes cranial nerve (CN) mononeuropathies—including CN3 (oculomotor), CN4 (trochlear), and CN6 (abducens)—as individual immune-related entities based on distinct clinical and MRI features. Analysis of OirAEs in lung cancer patients revealed that ophthalmoplegia represents one of the most prevalent manifestations (40.51%) [51]. All patients presenting with ophthalmoplegia were diagnosed with myasthenia gravis (MG), among which ptosis emerged as the predominant subtype. This ocular manifestation demonstrated a significant association with elevated mortality rates (23.33%). The onset of ophthalmoplegia typically occurred within the initial 10 weeks of ICI therapy and was associated with unfavorable prognosis.

Clinically, ophthalmoplegia is a hallmark symptom of metastatic central nervous system tumors, yet its potential induction by ICIs is frequently overlooked. Zahid et al. (2024) [72] demonstrated the clinical utility of the ice pack test (IPT) for early identification of ICI-mediated ocular weakness. Their study evaluated 14 patients who developed ptosis following ICI therapy, revealing IPT positivity (indicating ptosis improvement) in two myasthenia gravis cases, while the remaining 12 myositis patients showed negative IPT results.

### 5.2. Dry Eye

Dry eye disease represents a relatively common (prevalence ranging from 3% to 24%) yet frequently underestimated OirAE [5]. Although typically regarded as a mild complication, failure to promptly recognize and manage this condition may lead to patient discomfort, diminished quality of life, and potentially mask more severe OirAEs.

The pathogenesis of ICI-associated dry eye disease may involve disruption of ocular surface immune homeostasis by ICIs. ICIs potentially contribute to dry eye disease through the following mechanisms:

(a) T-cell-mediated inflammation: Activated autoreactive T cells may infiltrate conjunctival tissues, releasing proinflammatory cytokines that induce conjunctival inflammation.
(b) Glandular dysfunction: ICIs may target lacrimal and meibomian glands, resulting in reduced tear secretion and compromised tear film stability, thereby precipitating dry eye disease. Patrinely et al. [73] observed xerostomia as a frequent manifestation of chronic irAEs with an incidence rate of 2.4%, in their long-term follow-up study, suggesting that ICIs may impair glandular function, including that of the lacrimal glands.
(c) Autoantibody involvement: Although direct evidence remains limited, autoantibodies may theoretically attack conjunctival or lacrimal gland tissues.

### 5.3. Corneal Disorders

ICI-associated corneal manifestations may present in various forms, including keratitis, corneal ulceration, and even recurrent corneal erosion-like syndrome [70].

The pathogenesis of these corneal complications may involve several mechanisms:

(a) Direct immune-mediated attack: ICI-activated autoreactive T cells may directly target corneal cells, particularly epithelial cells, inducing inflammation and tissue damage. Since corneal epithelial cells express PD-L1, blockade of the PD-1/PD-L1 pathway could compromise corneal immune privilege, rendering it vulnerable to immune attack.
(b) Secondary to dry eye disease: Severe ICI-induced dry eye may lead to tear film instability and ocular surface desiccation, increasing susceptibility to corneal epithelial damage and secondary infections, thereby predisposing to keratitis or ulcer formation.
(c) Drug toxicity: Although ICIs primarily exert their effects through immunomodulation, direct cytotoxic effects on corneal cells or induction of proinflammatory mediators may also contribute to corneal pathology.

### 5.4. Retinopathy

ICI-associated retinopathies are relatively uncommon but can lead to significant visual impairment, including melanoma-associated retinopathy (MAR), cancer-associated retinopathy (CAR), and acute exudative paraneoplastic vitelliform maculopathy (AEPVM). Characteristic manifestations encompass painless vision loss, photopsia, and nyctalopia [40,41,74,75]. Retinal vasculitis, choroidal neovascularization, and serous retinal detachment may also occur, frequently coexisting with uveitis [74,76]. The pathogenic mechanisms of retinopathy may involve:

(a) Autoimmune targeting: The retina harbors numerous specific antigens, including retinal S-antigen and interphotoreceptor retinoid-binding protein (IRBP), which may become susceptible to attack by autoreactive T cells under ICI therapy. Retinal pigment epithelial (RPE) cells express PD-L1, and disruption of their immunosuppressive function by ICIs may precipitate RPE cell damage and retinal inflammation.
(b) Retinal vascular inflammation: ICIs may induce systemic vasculitis with retinal vascular involvement, leading to retinal vasculitis, vascular occlusion, and ischemic retinopathy. Au et al. (2023) [77] documented a case of nivolumab-associated peripheral vasculitis and digital gangrene, suggesting that ICIs could potentially trigger systemic vasculitis that may theoretically extend to retinal vasculature.
(c) Cytokine-mediated injury: Proinflammatory cytokines induced by ICIs may directly or indirectly compromise retinal cellular integrity, resulting in macular edema or retinal dysfunction.

## 6. Treatment Strategies

**Figure 4:**
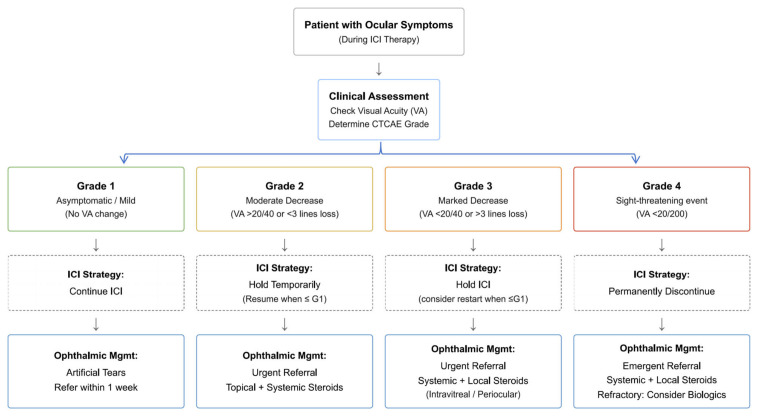
Step-by-step clinical management algorithm for ocular immune-related adverse events (irAEs). The flowchart integrates CTCAE grading severity with corresponding ICI treatment adjustments and ophthalmic interventions based on ASCO guidelines [78]. This algorithm provides a general CTCAE-based framework and should be modified for entity-specific conditions, such as optic neuritis and isolated dry eye disease, according to consensus recommendations and multidisciplinary assessment.

Our treatment recommendations follow the CTCAE 5.0 severity-stratified algorithm outlined in Figure 4, aligned with ASCO clinical guidelines for irAE management [78]. The risk–benefit balance of systemic immunomodulatory therapy in uveitis has been systematically evaluated [79], and these principles apply to OirAE management: efficacy in controlling inflammation must be weighed against potential adverse effects and the critical need to preserve antitumour immunity. We supplement this general framework with three key entity-specific updates from the 2025 international consensus on ocular irAE definitions [61]: (1) optic neuritis requires high-dose intravenous methylprednisolone (1000 mg/d × 3–5 d) regardless of CTCAE grade; (2) isolated dry eye disease can often be managed with topical therapy without ICI interruption, although severe ocular surface complications require individualized ophthalmic and oncologic assessment; and (3) permanent ICI discontinuation is no longer mandatory for Grade 3 events, with resumption permitted after inflammation resolves following multidisciplinary assessment.

Mechanistically targeted steroid-sparing agents may be considered in selected steroid-refractory cases. To provide clarity on the strength of supporting data, we propose the following evidence-level categorisation:

(i) Consensus-based recommendations (Grade C). Derived from the 2025 international consensus and ASCO irAE guidelines: local corticosteroids for Grade 1–2 events, systemic corticosteroids for Grade ≥ 3 events, and the ICI management algorithm shown in Figure 4.
(ii) Case-report-supported strategies (Grade D). Supported by published case reports or small case series in the OirAE setting, IL-6 receptor blockade (e.g., tocilizumab) has emerging supportive evidence in non-infectious uveitis and selected irAEs during anti-PD-1 therapy, but dedicated OirAE data remain limited to case reports. Anti-TNF-α therapy (infliximab, adalimumab) has been used for refractory ocular inflammation in the non-ICI setting; its use in ICI-associated uveitis is supported by isolated case reports and should be weighed carefully against the potential impact on systemic antitumour immunity.
(iii) Extrapolated from non-ICI uveitis practice. These strategies are supported by rigorous clinical trials or large observational studies in non-ICI uveitis populations. For adalimumab, a phase 3 randomized controlled trial (VISUAL I) [80] demonstrated efficacy in active non-infectious intermediate, posterior, and panuveitis. For tocilizumab, retrospective data in Birdshot retinochoroiditis and refractory non-anterior uveitis suggest potential benefit, but controlled trials in the ICI setting are lacking [81,82].
(iv) Speculative mechanism-based therapies. IL-17A inhibition, JAK inhibitors, and FcRn inhibition are biologically plausible based on the mechanistic framework outlined in Section 3, but clinical evidence in OirAEs is currently absent. These approaches require prospective evaluation before clinical recommendation.

## 7. Future Research Directions and Challenges

A primary challenge in managing severe OirAEs lies in the fundamental conflict associated with current standard treatments: systemic corticosteroids, while effective for ocular inflammation, pose a theoretical risk of dampening the T-cell-mediated anti-tumor response critical for patient survival. Future research should therefore prioritize therapeutic strategies that may better control ocular autoimmunity while minimizing interference with systemic anti-tumor immunity. Instead of broad-spectrum immunosuppression, clinical trials should rigorously evaluate steroid-sparing biologics, such as IL-6 inhibitors (e.g., tocilizumab), which may offer a more favorable safety profile in oncology settings by targeting specific inflammatory pathways without broadly impeding cytotoxic T-lymphocyte activity [81,82].

Furthermore, the management of ICI-related ocular toxicity must evolve from a reliance on generic clinical grading systems toward molecular phenotyping. Current guidelines treat ICI-uveitis as a monolithic entity, yet its pathogenesis is mechanistically heterogeneous. Future investigations should focus on validating predictive biomarkers in tear fluid or aqueous humor, such as specific cytokine signatures, to distinguish between T-cell-driven and autoantibody-mediated phenotypes. This shift would enable a precision medicine approach, allowing clinicians to select targeted therapies (e.g., JAK inhibitors) tailored to the specific immunopathogenesis of the individual patient.

Finally, expanding the therapeutic arsenal requires exploring mechanisms beyond direct T-cell suppression. Novel approaches, such as neonatal Fc receptor (FcRn) inhibition, represent a paradigm shift for antibody-mediated conditions like myasthenia gravis, potentially clearing pathogenic autoantibodies without suppressing the cellular immunity required for tumor control [83]. Concurrently, advancements in local drug delivery, such as suprachoroidal injections, offer the promise of achieving high intraocular drug concentrations with negligible systemic absorption [84]. Integrating these targeted, mechanism-based strategies will be essential to preserving visual function while maintaining the efficacy of life-saving cancer immunotherapies.

## 8. Conclusions

OirAEs encompass a heterogeneous spectrum of immune-mediated toxicities affecting nearly all anatomical compartments of the eye, ranging from mild dry eye disease to sight-threatening uveitis, optic neuritis and orbital inflammation. The recent 2025 international consensus classification of 19 distinct OirAE entities has established a standardized framework that resolves long-standing inconsistencies in diagnosis and reporting across clinical and research settings. In this review, we have integrated this consensus classification to provide a systematic overview of OirAE pathogenic mechanisms, clinical phenotype spectrum, and consensus-based and evidence-informed management strategies. The molecular pathogenesis of OirAEs may involve tissue-specific disruption of ocular immune privilege, heterogeneous T-cell subset activation, and compartment-specific cytokine release, with distinct pathways potentially contributing to damage in different ocular structures: CD8+ cytotoxic T cells targeting shared tumor–ocular melanocyte antigens may contribute to uveitis, Th17-skewed responses may participate in ocular surface disease, and Th1/IFN-γ activity has been implicated in neuro-ophthalmic toxicities. Optimal OirAE management requires close multidisciplinary collaboration between oncologists and ophthalmologists. Early diagnosis and individualized, phenotype-specific treatment strategies may help preserve visual function while minimizing unnecessary interruption of effective cancer therapy. Future research priorities include validation of tear/aqueous humor cytokine biomarkers for patient stratification, development of mechanism-based targeted therapies that better control ocular autoimmunity while minimizing interference with systemic anti-tumor immunity, and establishment of prospective registries using the 2025 consensus diagnostic criteria to expand evidence for rare OirAE phenotypes. The ultimate goal of OirAE management is to transition from reactive, generalized immunosuppression to proactive, precision medicine approaches that preserve visual function while maintaining the efficacy of life-saving cancer immunotherapy. This review provides a comprehensive, consensus-aligned reference for clinicians to improve recognition and management of these underrecognized toxicities.

## Figures and Tables

**Table 1:** Classes of approved immune checkpoint inhibitors.

Class	Drug Name	Initial Approved Indication	First Approved Year	Regional Approval (as of 2026)
CTLA-4 inhibitors	Ipilimumab (Yervoy)	Melanoma [13]	2011	US, EU, JP
	Tremelimumab (Imjudo)	Hepatocellular carcinoma (in combination with durvalumab) [14]	2022	US, EU, JP
PD-1 inhibitors	Nivolumab (Opdivo)	Melanoma [15]	2014	US, EU, JP, CN
	Pembrolizumab (Keytruda)	Melanoma [16]	2014	US, EU, JP, CN
	Cemiplimab (Libtayo)	Cutaneous squamous cell carcinoma [17]	2018	US, EU
	Toripalimab (Tuoyi)	Melanoma (China-approved) [18]	2018	CN
	Dostarlimab (Jemperli)	Endometrial cancer (dMMR) [19]	2021	US, EU
PD-L1 inhibitors	Atezolizumab (Tecentriq)	Urothelial carcinoma [20]	2016	US, EU, JP, CN
	Avelumab (Bavencio)	Merkel cell carcinoma [21]	2017	US, EU
	Durvalumab (Imfinzi)	Urothelial carcinoma [22]	2017	US, EU, JP, CN
	Envafolimab	MSI-H/dMMR solid tumors (China-approved, first subcutaneous PD-L1) [23]	2021	CN
	Sugemalimab	NSCLC (chemotherapy combination) (China-approved) [24]	2021	CN, EU
LAG-3 inhibitors	Relatlimab (Opdualag)	Melanoma (combination with nivolumab) [25]	2022	US, EU, JP

CTLA-4, cytotoxic T-lymphocyte-associated protein 4; LAG-3, lymphocyte-activation gene 3; PD-1, programmed cell death protein 1; PD-L1, programmed death-ligand-1. Approval status is continuously evolving; this table reflects approvals documented as of mid-2026.

**Table 2:** Clinical features of ICI-related OirAEs.

OirAE Type	Clinical Presentation	Diagnostic Approach	Representative References
Uveitis (Anterior)	Ocular pain, photophobia, redness, decreased vision, keratic precipitates, anterior chamber cells/flare	Slit-lamp examination, intraocular pressure (IOP) measurement	[62,66,69]
Uveitis (Intermediate/Posterior/Panuveitis)	Floaters, decreased vision, vitritis, chorioretinal lesions, serous retinal detachment (VKH-like)	Dilated fundoscopy, optical coherence tomography (OCT), fluorescein angiography (FA), indocyanine green angiography (ICGA), B-scan ultrasonography	[62,64,66]
Ocular Myopathy/Extraocular Muscle Inflammation	Diplopia, ptosis, limited eye movement, ocular pain	Neuro-ophthalmologic exam, ice pack test (IPT), creatine kinase (CK), autoantibodies, repetitive nerve stimulation (RNS)/single-fiber electromyography (SFEMG), orbital MRI/CT	[27,51]
Conjunctivitis	Eye redness, foreign body sensation, tearing, discharge, itching	Slit-lamp examination (conjunctival hyperemia, edema, follicles)	[51,70]
Dry Eye Disease	Ocular dryness, burning sensation, foreign body sensation, photophobia, fluctuating vision	Tear break-up time, Schirmer test, corneal staining	[51,70]
Corneal Disorders	Eye pain, photophobia, blurred vision, corneal inflammation/ulceration/erosion	Slit-lamp examination (corneal epithelial/stromal lesions), corneal staining	[70]
Retinal Disorders	Decreased vision, metamorphopsia, visual field defects, photopsia	Visual acuity, fundus examination, OCT, FA, electroretinography (ERG)	[27,51]
Optic Neuritis	Acute visual loss, visual field defects, eye pain, color vision defects	Neuro-ophthalmologic examination, relative afferent pupillary defect (RAPD), visual evoked potential (VEP), orbital MRI	[51]
Orbital Inflammation/Orbital Lesions	Proptosis, eye pain, eyelid edema, diplopia, decreased vision	Ophthalmic examination, orbital CT/MRI, blood inflammatory markers, orbital biopsy	[51,70]

IOP: intraocular pressure; OCT: optical coherence tomography; FA: fluorescein angiography; ICGA: indocyanine green angiography; IPT: ice pack test; CK: creatine kinase; RNS: repetitive nerve stimulation; SFEMG: single-fiber electromyography; MRI: Magnetic Resonance Imaging; CT: Computed Tomography; RAPD: relative afferent pupillary defect; VEP: visual evoked potential; ERG: electroretinography.

## Data Availability

No new data were created or analyzed in this study. Data sharing is not applicable to this article.

## Abbreviations

AEPVM	Acute Exudative Paraneoplastic Vitelliform Maculopathy
APCs	Antigen-Presenting Cells
ASCO	American Society of Clinical Oncology
CAR	Cancer-Associated Retinopathy
CK	Creatine Kinase
CT	Computed Tomography
CTCAE	Common Terminology Criteria for Adverse Events
CTLA-4	Cytotoxic T-Lymphocyte-Associated Protein 4
CXCL10	C-X -C Motif Chemokine Ligand 10
ERG	Electroretinography
FDA	U.S. Food and Drug Administration
FcRn	Neonatal Fc Receptor
FA	Fluorescein Angiography
G-CSF	Granulocyte Colony-Stimulating Factor
ICGA	Indocyanine Green Angiography
ICIs	Immune Checkpoint Inhibitors
IFN-γ	Interferon-Gamma
IL-2	Interleukin-2
IL-6	Interleukin-6
IRBP	Interphotoreceptor Retinoid-Binding Protein
IPT	Ice Pack Test
irAEs	Immune-Related Adverse Events
IOP	Intraocular Pressure
LAG-3	Lymphocyte-Activation Gene 3
MAR	Melanoma-Associated Retinopathy
MG	Myasthenia Gravis
MRI	Magnetic Resonance Imaging
NSCLC	Non-Small Cell Lung Cancer
OCT	Optical Coherence Tomography
OR	Odds Ratio
OirAEs	Ocular Immune-Related Adverse Events
PD-1	Programmed Cell Death Protein 1
PD-L1	Programmed Death-Ligand 1
RAPD	Relative Afferent Pupillary Defect
RPE	Retinal Pigment Epithelium
RNS	Repetitive Nerve Stimulation
SFEMG	Single-Fiber Electromyography
TIM-3	T-Cell Immunoglobulin and Mucin-Domain Containing-3
TIGIT	T-Cell Immunoglobulin and ITIM Domain
TNF-α	Tumor Necrosis Factor-Alpha
Tregs	Regulatory T Cells
TRP-1	Tyrosinase-Related Protein-1
VISTA	V Domain Ig Inhibitory Factor Activated by T Cells
VKH	Vogt–Koyanagi–Harada Syndrome
VSIG-3	V-Set and Immunoglobulin Domain Containing 3
VEP	Visual Evoked Potential
